# Identifying priorities for medication management resources for people living with dementia and their care givers through community action

**DOI:** 10.1002/alz.085810

**Published:** 2025-01-09

**Authors:** Karen Watson, Amanda Cross, Natali Jokanovic, Jacqueline Wesson, Joanne Lo, Danijela Gnjidic, Mouna J Sawan

**Affiliations:** ^1^ The University of Sydney, Camperdown, NSW Australia; ^2^ Monash University, Parkville, VIC Australia; ^3^ Monash University, Melbourne, VIC Australia; ^4^ University of Sydney, Camperdown, NSW Australia

## Abstract

**Background:**

People with dementia and their care givers are provided limited guidance in medication management, potentially contributing to medication‐related harm. Importantly, there are no resources that provide comprehensive medication management guidance across care settings. To ensure that resources are co‐designed, genuine involvement of people with dementia, their care givers and the community in identifying the priorities for medication management guidance resources is needed. We explored community‐centred priorities for medication management guidance resources for people with dementia and their care givers.

**Methods:**

We established a 23‐member consortium partnership with people living with dementia, care givers, healthcare professionals, and national consumer and professional organisations using a community‐based participatory research approach. A qualitative descriptive design, using four focus groups and two interviews with key informants was conducted between September to December 2023 to explore partners’ priorities for medication management resources across care settings. Content analysis was performed to generate a list of priorities.

**Results:**

The key priorities for inclusion in the medication resource were to: 1) empower the person with dementia and their care givers to make informed choices, by providing pragmatic and accessible information to meet their needs and promote key questions to engage health providers in shared decision‐making; 2) inclusion of strategies to address medication challenges, and 3) suggested format and setting for the availability of information.

**Conclusion:**

This is the first time a community action approach has been adopted to co‐design resources to support people with dementia and care givers in medication management challenges. The community‐centred priorities will be used to generate an inventory of consumer‐tailored communication strategies for people with dementia and their care givers.

